# Supplementary material for: First Description of a Temperate Bacteriophage (vB_FhiM_KIRK) of Francisella hispaniensis Strain 3523
Source: Viruses. 2021 Feb 20;13(2):327. doi: 10.3390/v13020327 (PMC7924643; doi:10.3390/v13020327)
Supplement: Supplementary file 1 [file viruses-13-00327-s001.pdf]

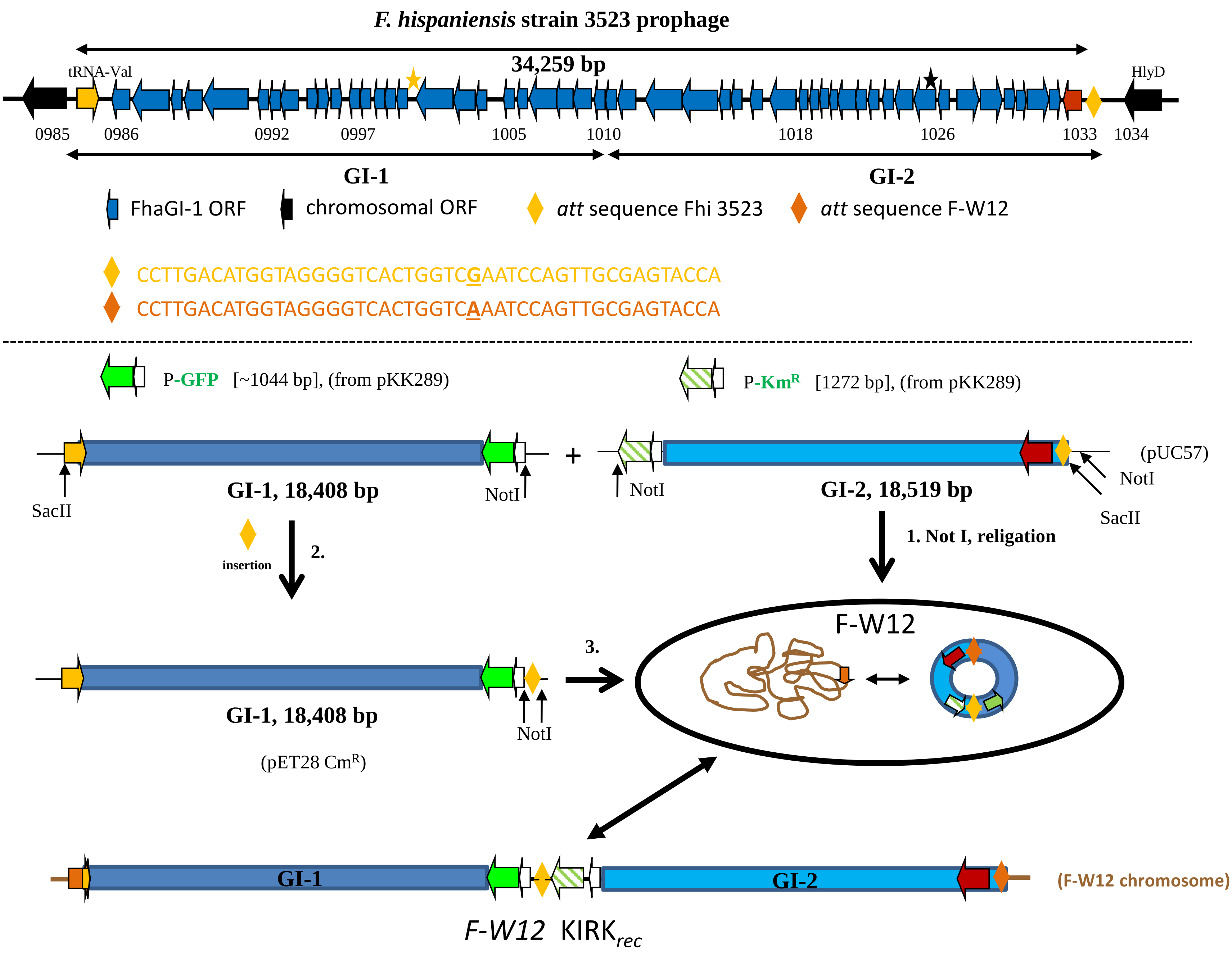

F. hispaniensis strain 3523 prophage
34,259 bp
tRNA-Val
HlyD
0985
0986
0992
0997
1005
1010
1018
1026
1033
1034
GI-1
GI-2
FhaGI-1 ORF
chromosomal ORF
att sequence Fhi 3523
att sequence F-W12
CCTTGACATGGTAGGGGTCACTGGTCGAATCCAGTTGCGAGTACCA
CCTTGACATGGTAGGGGTCACTGGTCAAATCCAGTTGCGAGTACCA
P-GFP [~1044 bp], (from pKK289)
P-KmR [1272 bp], (from pKK289)
SacII
GI-1, 18,408 bp
NotI
+
NotI
GI-2, 18,519 bp
(pUC57)
NotI
SacII
insertion
2.
1. Not I, religation
F-W12
3.
GI-1, 18,408 bp
NotI
(pET28 CmR)
GI-1
GI-2
(F-W12 chromosome)
F-W12 KIRKrec

Supplemental Figure 1. Cloning strategy of the recombinant bacteriophage $KIRK_{rec}$.

DNA sequences of GI-1 and GI-2 were synthesized by GeneCust (GeneCust, Dudelang, Luxembourg). GI-1 contains the genes tRNA-Val to FN3523_1010 followed by a *gfp* gene with promotor from vector pKK289KmGFP (Bönquist et al., 2008) and in GI-2 the KmR casette from pKK289 is followed by genes FN3523_1011 to 1033, including the *att* (*attR*) site. Cloning of the complete phage *in vitro* was not possible and therefore we changed the cloning strategy using the recombinase activity of FN3523_1033 (Rydzewski et al., 2015): (1.) GI-2 was cut out with the restricion enzyme NotI, religated and electroporated into *Francisella* sp. strain W12-1067 (*F*-W12) it integrated site-specifically into the tRNA-Val gene of *F*-W12 (exhibiting one SNP compared to *Fhi* 3523) through the activity of gene FN3533-1033). (2.) We cloned a second *att* site into the 3‘-end of the DNA fragment of GI-1 and (3.) this construct was electroporated into *F*-W12 harbouring GI-2. (4.) PCR analysis and DNA sequencing revealed that the strategy was successful in generating a F-W12 strain harbouring a complete $KIRK_{rec}$ prophage within its genome. PCR analysis revealed that all three episomal forms of $KIRK_{rec}$ (GI-1 part, GI-2 part [both episomal forms are not given in the figure] and whole $KIRK_{rec}$) are present in *F*-W12/$KIRK_{rec}$ (data not shown).

Bönquist, L., Lindgren, H., Golovliov, I., Guina, T., and Sjostedt, A. MglA and Igl proteins contribute to the modulation of Francisella tularensis live vaccine strain-containing phagosomes in murine macrophages. Infect Immun 2008, 76, 3502-3510.

Rydzewski, K.; Tlapak, H.; Niehaus, I.P.; Dabrowski, P.W.; Grunow, R.; Heuner, K. Identification and characterization of episomal forms of integrative genomic islands in the genus Francisella. Int J Med Microbiol 2015, 305, 874-880, doi:10.1016/j.ijmm.2015.08.037.

Supplemental Table 1: Primers used in this study.

Primer	Tm [°C]	Sequence 5`--> 3`	Reference
F1_out_U	58.9	tgcgcgacacagaataatatctc	this work
F2_out_R	58.9	ctgttgattttcctacagtcgct	this work
F2_out_U	56	tagccatacgaagcttgacattat	this work
F3_out_R	56	acatacgatgactcattagaagca	this work
Fha-1	61.9	aatcactccaatagccagtactaagga	Rydzewski et al., 2015
Fha-1^{W12}	58.9	cttgcttcaatgactgggttttg	Tlapak et al. 2018
Fha-2	61.6	cttgtcaacaactggtctactttctc	Rydzewski et al., 2015
Fha-3	59.3	ctgagaattaagccacttatatcagaat	Rydzewski et al., 2015
Fha-4	60.4	atactctacacaaggatggtgaaacat	Rydzewski et al., 2015
Fha-4* (Fth-specific)	57.1	gtaaaacccgttggtcaaccttatcag	Rydzewski et al., 2015
Fha-4^{W12}	58.9	atccaggaatctttgtaggagct	Tlapak et al. 2018
Fha996_U	57.9	ttgtaattggtcgtgagcctg	this work
Fha997_R	55.8	tagaattaccttttaatcttaaattattatatc	this work
Fhis_R13	57.9	gctaggacttctgcaaagtag	this work
Fhis_U13A	56.6	gatctagctggagtactgc	this work
